# Supplementary material for: A Critical View of the Application of the APEX Software (Aqueous Photochemistry of Environmentally-Occurring Xenobiotics) to Predict Photoreaction Kinetics in Surface Freshwaters
Source: Molecules. 2019 Dec 18;25(1):9. doi: 10.3390/molecules25010009 (PMC7017383; doi:10.3390/molecules25010009)
Supplement: Supplementary file 1 [file molecules-25-00009-s001.zip › 3.2.4_gcc-4.4.0.zip!/3.2.4_gcc-4.4.0/3.2.4_gcc-4.4.0/doc/octave/html/interpreter/Single-Line-Comments.html]

Single Line Comments - Untitled


Next: Block Comments,
Up: Comments


---

#### 2.7.1 Single Line Comments

In the Octave language, a comment starts with either the sharp sign
character, #, or the percent symbol % and continues to the
end of the line. Any text following the sharp sign or percent symbol is
ignored by the Octave interpreter and not executed. The following example
shows whole line and partial line comments.

```
     function countdown
       # Count down for main rocket engines
       disp(3);
       disp(2);
       disp(1);
       disp("Blast Off!");  # Rocket leaves pad
     endfunction
```
